# DeepStrain Evidence of Asymptomatic Left Ventricular Diastolic and Systolic Dysfunction in Young Adults With Cardiac Risk Factors

**DOI:** 10.3389/fcvm.2022.831080

**Published:** 2022-04-11

**Authors:** Manuel A. Morales, Gert J. H. Snel, Maaike van den Boomen, Ronald J. H. Borra, Vincent M. van Deursen, Riemer H. J. A. Slart, David Izquierdo-Garcia, Niek H. J. Prakken, Ciprian Catana

**Affiliations:** ^1^Department of Radiology, Athinoula A. Martinos Center for Biomedical Imaging, Massachusetts General Hospital and Harvard Medical School, Boston, MA, United States; ^2^Harvard-MIT Division of Health Sciences and Technology, Cambridge, MA, United States; ^3^Department of Radiology, Medical Imaging Center, University Medical Center Groningen, University of Groningen, Groningen, Netherlands; ^4^Cardiovascular Research Center, Massachusetts General Hospital and Harvard Medical School, Boston, MA, United States; ^5^Department of Nuclear Medicine and Molecular Imaging, Medical Imaging Center, University Medical Center Groningen, University of Groningen, Groningen, Netherlands; ^6^Department of Cardiology, University Medical Center Groningen, University of Groningen, Groningen, Netherlands; ^7^Department of Biomedical Photonic Imaging, Faculty of Science and Technology, University of Twente, Enschede, Netherlands

**Keywords:** deep learning, myocardial strain, young adults, risk factors, cardiac MRI, left ventricular dysfunction

## Abstract

**Purpose:**

To evaluate if a fully-automatic deep learning method for myocardial strain analysis based on magnetic resonance imaging (MRI) cine images can detect asymptomatic dysfunction in young adults with cardiac risk factors.

**Methods:**

An automated workflow termed DeepStrain was implemented using two U-Net models for segmentation and motion tracking. DeepStrain was trained and tested using short-axis cine-MRI images from healthy subjects and patients with cardiac disease. Subsequently, subjects aged 18–45 years were prospectively recruited and classified among age- and gender-matched groups: risk factor group (RFG) 1 including overweight without hypertension or type 2 diabetes; RFG2 including hypertension without type 2 diabetes, regardless of overweight; RFG3 including type 2 diabetes, regardless of overweight or hypertension. Subjects underwent cardiac short-axis cine-MRI image acquisition. Differences in DeepStrain-based left ventricular global circumferential and radial strain and strain rate among groups were evaluated.

**Results:**

The cohort consisted of 119 participants: 30 controls, 39 in RFG1, 30 in RFG2, and 20 in RFG3. Despite comparable (>0.05) left-ventricular mass, volumes, and ejection fraction, all groups (RFG1, RFG2, RFG3) showed signs of asymptomatic left ventricular diastolic and systolic dysfunction, evidenced by lower circumferential early-diastolic strain rate (<0.05, <0.001, <0.01), and lower septal circumferential end-systolic strain (<0.001, <0.05, <0.001) compared with controls. Multivariate linear regression showed that body surface area correlated negatively with all strain measures (<0.01), and mean arterial pressure correlated negatively with early-diastolic strain rate (<0.01).

**Conclusion:**

DeepStrain fully-automatically provided evidence of asymptomatic left ventricular diastolic and systolic dysfunction in asymptomatic young adults with overweight, hypertension, and type 2 diabetes risk factors.

## Introduction

Worldwide shifts toward sedentary lifestyles and suboptimal diets have led to increased prevalence of global obesity and obesity-related comorbidities such as hypertension (1, 2). Both conditions alone are strong independent risk factors for type 2 diabetes mellitus (T2DM) (3), and increased cardiovascular morbidity and mortality (4). Obesity, hypertension, and T2DM interact synergistically to influence cardiac remodeling, resulting in a markedly heightened risk of cardiovascular disease and heart failure when these risk factors co-cluster (5). Further, links have been made between these risk factors and the increased incidence of heart failure in the young (6). The manifestations of heart failure could be preceded by asymptomatic left ventricular diastolic dysfunction (ALVDD), a silent disease whose development and progression are stimulated by comorbidities, specially obesity, hypertension, and T2DM (7). Strain and strain rate (SR) imaging have emerged as promising technologies for the assessment of myocardial deformation and earlier detection of asymptomatic dysfunction. Indeed, a recent echocardiography strain study reported ALVDD in young adults with obesity, as evidenced by reduced diastolic SR (8). Other studies in older adults have shown that asymptomatic left ventricular systolic dysfunction (ALVSD) can coexist with ALVDD even when ejection fraction is preserved, and this is associated with adverse long-term prognosis (9, 10). Thus, strain imaging tools could provide a sensitive and comprehensive evaluation of left ventricular (LV) function complementary to ejection fraction. Further, identification of both ALVDD and ALVSD at the onset of non-specific symptoms could represent the earliest opportunity for diagnosis and treatment (7, 9).

Echocardiographic examinations can be non-diagnostic or have inconclusive findings, especially in obese patients (11). In such cases, cardiac magnetic resonance imaging (MRI) plays an important complementary role and is often requested as it provides the most accurate and reproducible assessment of cardiac function, structure, and myocardial tissue properties (12). Cardiac MRI feature tracking methods requiring only clinically standard cine images could offer advanced strain readouts as well, providing additional information about the underlying biomechanical motion without modifications to the imaging protocol. In addition, cine-based strain analysis has been used to detect ALVDD and ALVSD in older adults (13–15). Nevertheless, feature tracking is a time-consuming process that requires considerable training and expertise to perform. Semi-automatic methods are available in commercial software. However, manual correction of tracked myocardial boundaries is still often required, which results in operator-related discrepancies in strain measures (16). Alternatively, we have shown that myocardial deformation can be automatically quantified from short-axis cine-MRI images using convolutional neural networks (17). Further, we have developed and validated a deep learning method for fully-automatic strain analysis (i.e., DeepStrain) based on cine-MRI images (18). DeepStrain showed significant differences among patients with known cardiac disease. However, characterization of asymptomatic dysfunction is potentially more difficult compared to older populations, since they are less likely to have obvious clinical or imaging signs of cardiac disease during examination (19). Indeed, few cardiac MRI strain studies have focused on younger populations with cardiac risk factors, yet identification and characterization of ALVDD and ALVSD in this population is of clinical and public health importance since young adults have a life-time risk of heart failure as high as 20%, and may benefit the most from earlier treatment (20).

Thus, we sought to evaluate if a fully-automatic deep learning method for myocardial strain analysis based on cardiac cine-MRI can detect asymptomatic dysfunction in asymptomatic young adults with overweight, hypertension, and T2DM cardiac risk factors.

## Materials and Methods

### Study Population

**Figure 1:**
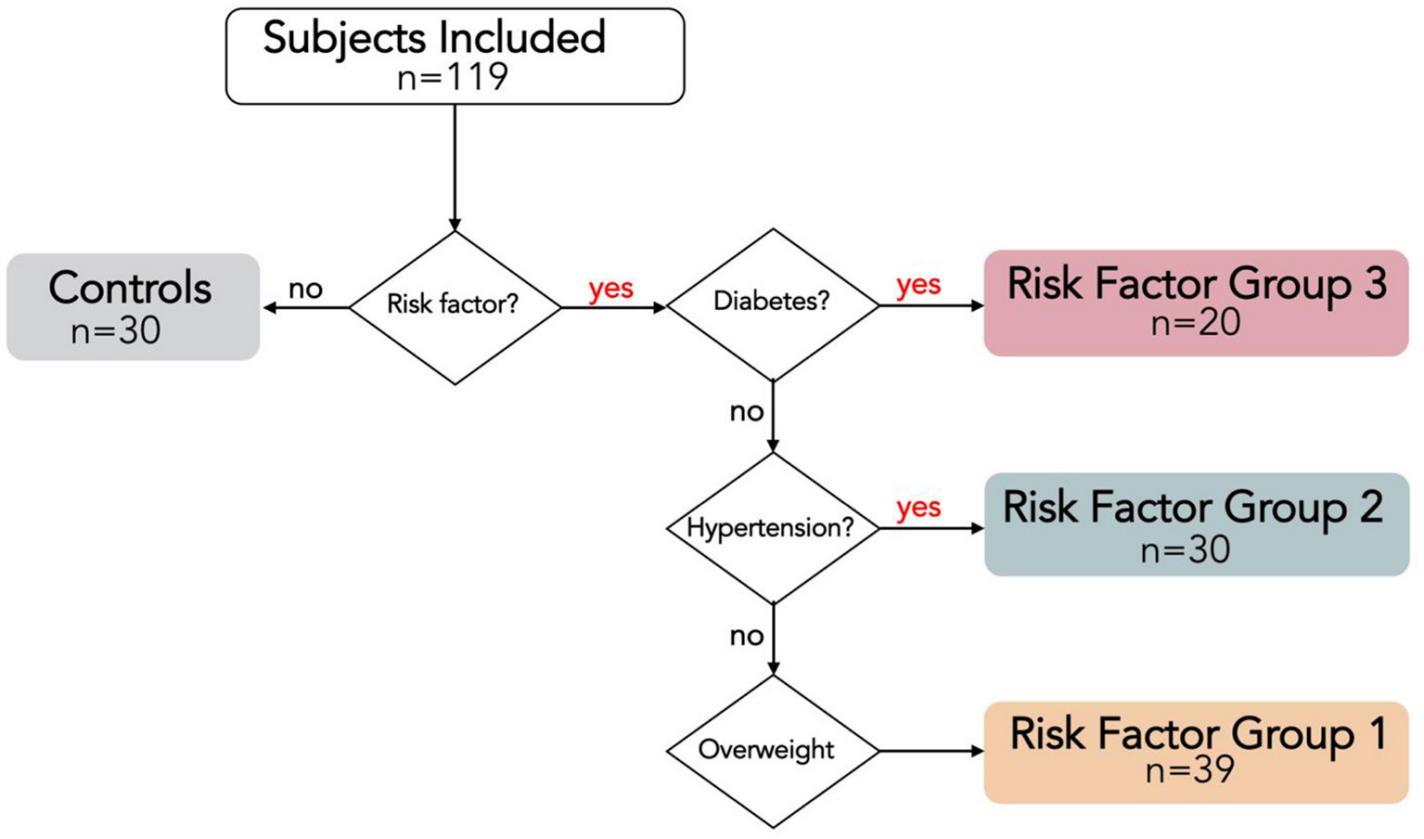
Risk Factor Group (RFG) classifications visualized with a flow chart. RFG3 includes all type 2 diabetes mellitus (T2DM) subjects, regardless of the presence of hypertension or overweight. RFG2 includes all hypertensive subjects without T2DM, regardless of the presence of overweight. RGF1 includes all overweight subjects without hypertension and T2DM.

This prospective cross-sectional single-center study was approved by the local medical ethical committee, and conducted in accordance with the Declaration of Helsinki. Subjects aged 18–45 years were voluntarily recruited with public advertisements and signed informed consent before participation. Exclusion criteria were history or knowledge of cardiac disease, cardiac risk factors other than overweight, hypertension or T2DM, exercising >3 h/week (21), and contraindications to cardiac MRI. Presence of cardiac risk factors was checked using a medical questionnaire, and with measurements of weight, blood pressure and hemoglobin A1c (HbA1c). Overweight was defined as body mass index (BMI) ≥ 25 kg/m^2^; hypertension was defined as either actively under pharmacological treatment or three consecutive blood pressure measurements ≥ 140/90 mmHg; T2DM was defined as either actively under pharmacological treatment or a HbA1c level ≥ 48 mmol/mol measured prior to the MRI exam. All subjects were classified into one of the following age- and gender-matched groups: controls including all subjects without risk factors; risk factor group 1 (RFG1) including all overweight subjects with neither hypertension nor T2DM; RFG2 including all hypertensive subjects without T2DM, regardless of the presence or absence of additional overweight; RFG3 including all subjects with T2DM, regardless of the presence or absence of additional overweight or hypertension (Figure 1).

### MRI Acquisition

All subjects underwent cardiac MRI using a 3 Tesla (T) scanner (MAGNETOM Prisma, Siemens Healthineers, Erlangen, Germany) with a 60-element phased-array body coil. Experienced operators used a retrospective electrocardiogram (ECG)-gated steady-state free precession sequence to acquire short-axis cines covering the heart from atria to ventricular apex during a series of breath-holds. Each cine contained 25 cardiac phases with 6 mm slice thickness, and 4 mm interslice gaps. Short-axis acquisition parameters were: repetition time 38.92–44.52 ms, echo time 1.15–1.31 ms, flip angle 43–46°, field of view 300–453 × 225–453 mm^2^, acquisition matrix 256 × 192–256.

### DeepStrain Analysis

**Figure 2:**
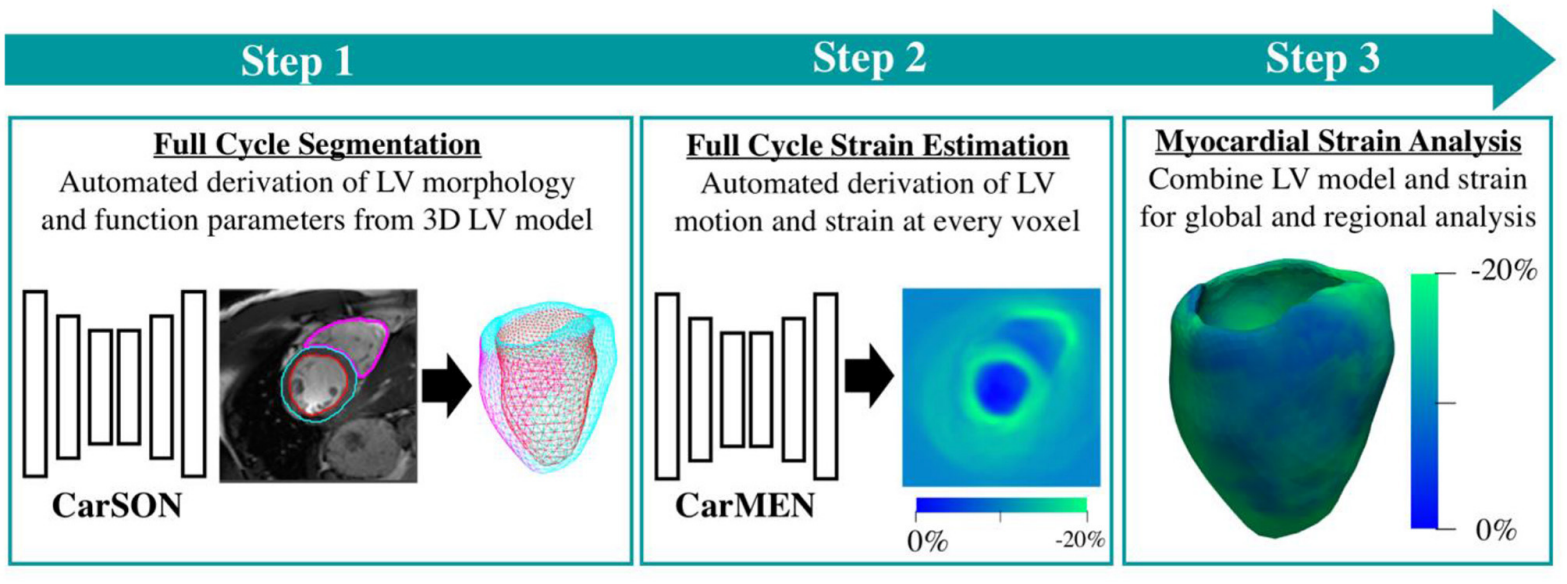
DeepStrain workflow. The first step is segmentation of the left and right ventricular tissues. The second step is evaluation of left-ventricular motion. These two steps are performed using deep learning convolutional neural networks. These networks are combined into a single workflow for fast and automated strain analysis (third step).

The DeepStrain workflow uses a pair of cine-MRI stacks at two different time frames (Figure 2). A cardiac segmentation network (CarSON) generated tissue labels for a single time frame. This was done for all time frames, and labels were used to calculate LV morphology and function. Since Lagrangian strain evaluates myocardial deformation with the end-diastolic phase as the reference, end-diastolic labels were used to construct the LV anatomical model required for strain analysis. A cardiac motion estimation network (CarMEN) was used to generate motion vectors using a pair of time frames concatenated along the channel dimension, which were used to calculate the Lagrangian strain (17). The first time frame was kept always at end-diastole, while the second frame was evaluated for all contracted phases. Per-voxel strain measures were combined with the LV labels to extract both global and regional circumferential and radial strain values. We evaluated global end-systolic strain, global systolic and early-diastolic SR, and regional end-systolic strain for each ventricular wall according to the American Heart Association polar map (22), i.e., anterior (regions 1, 7, and 13), septal (regions 2, 3, 8, 9, and 14), inferior (regions 4, 10, and 15), and lateral (regions 5, 6, 11, 12, and 16). The apex region 17 was excluded from the regional analysis but included in the global evaluation. The combined DeepStrain workflow was trained using short-axis cine-MRI data that was obtained using 1.5 T (Siemens Area, Siemens Medical Solutions, Germany) and 3.0 T scanners (Siemens Trio Tim, Siemens Medical Solutions, Germany). The data was collected from 150 subjects evenly divided into five groups, including healthy subjects and patients with hypertrophic cardiomyopathy, abnormal right ventricle, myocardial infarction with reduced ejection fraction, and dilated cardiomyopathy (23). DeepStrain automatically processed the data from each subject (~25 frames) in <2.2 min on a 32GB RAM CPU. The model and pre-trained parameters were made freely available online.

### Statistical Analysis

Demographic variables were expressed as mean ± standard deviation, and strain-related variables as mean [95% confidence interval]. Variables were tested for normality with a Shapiro-Wilk test and for homogeneity of variance using Levene's test. One-way analysis of variance (ANOVA) with *post-hoc* test by Bonferroni was used to examine differences among groups. Multivariate linear regression analysis was used to identify the independent association of the clinical variables body surface area, mean arterial pressure, and HbA1c on global strain measures. A *p*-value <0.05 was considered statistically significant. Data were analyzed using Python (version 3.5, Python Software Foundation, www.python.org).

## Results

The study cohort consisted of 119 participants (35 ± 5 years, 50% male) including the control group with 30 subjects; RFG1 with 39 overweight subjects; RFG2 with 30 hypertensive subjects, including 13 (43%) with additional overweight; RFG3 with 20 T2DM subjects, including 11 (55%) with additional overweight, 1 (5%) with additional hypertension and 8 (40%) with both (Table 1). The ECG recorded during cardiac MRI showed regular cardiac rhythm in all subjects. Mean heart rate of RFG3 was significantly higher compared to controls (*p* < 0.001). Furthermore, there were no significant differences between groups in LV mass, volumes, and ejection fraction (Supplementary Table 1).

**Table 1:** Clinical characteristics of controls and risk factor groups (RFG).

	**Controls**	**RFG1**	**RFG2**	**RFG3**	***P*-value**
	**(*n* = 30)**	**(*n* = 39)**	**(*n* = 30)**	**(*n* = 20)**	
Age (years)	34 ± 3	33 ± 6	36 ± 5	37 ± 5	0.051
Gender, male	16 (53)	19 (49)	14 (47)	10 (50)	0.964
Height (cm)	178 ± 7	175 ± 8	178 ± 10	174 ± 10	0.206
Weight (kg)	70 ± 9	94 ± 13^***^	82 ± 16^*^	98 ± 19^***^	<0.001
Body mass index (kg/m^2^)	22 ± 2	30 ± 3^***^	25 ± 5^***^	32 ± 5^***^	<0.001
Body surface area (m^2^)	1.9 ± 0.2	2.1 ± 0.2^***^	2.0 ± 0.2	2.2 ± 0.3^***^	<0.001
Heart rate (bpm)	61 ± 9	67 ± 11	68 ± 8	77 ± 8^***^	<0.001
Systolic blood pressure (mmHg)	117 ± 8	122 ± 8	138 ± 18^‡^	136 ± 12^***^	<0.001
Diastolic blood pressure (mmHg)	78 ± 5	81 ± 6	92 ± 11^‡^	89 ± 8^***^	<0.001
Hemoglobin A1c (mmol/mol)	33 ± 3	34 ± 3	32 ± 3	61 ± 17^***^	<0.001
Hypertension medication, yes	–	–	24 (80)	4 (20)	–
Type 2 diabetes medication, yes	–	–	–	20 (100)	–

*Data is presented as either mean ± standard deviation or number (percentage). P-value shows the one-way ANOVA test value between groups. post-hoc test by Bonferroni*:

* *p <0.05*;

*** *p <0.001 vs. controls*.

### Global Strain and Strain Rate

**Figure 3:**
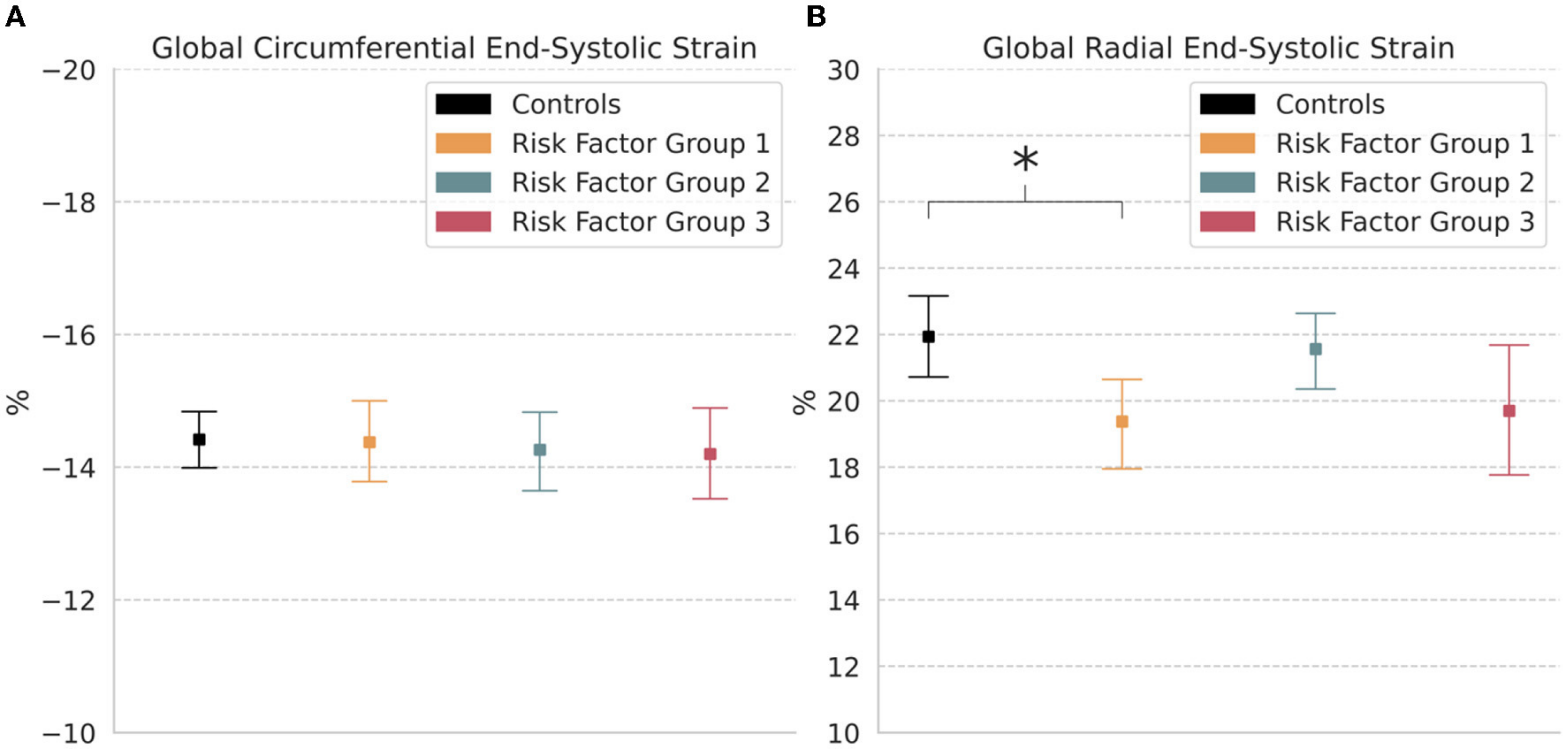
Global strain. Measures of **(A)** Circumferential and **(B)** Radial end-systolic strain in controls and risk factor groups. Strain results are visualized as mean with 95% confidence interval. *post-hoc* test by Bonferroni: *p < 0.05.

Comparisons of global end-systolic strain showed that there were no significant differences in circumferential and radial strains between groups, except for a significantly decreased radial strain in RFG1 (19.4% [18.0, 20.8]; *p* < 0.05) relative to controls (21.9% [20.7, 23.1]) (Table 2; Figure 3).

**Table 2:** Left-ventricular strain in controls and risk factor groups (RFG).

	**Controls**	**RFG1**	**RFG2**	**RFG3**	***P*-value**
**Circumferential**					
Global end-systolic strain (%)	−14.4 [−14.9, −14.0]	−14.4 [−15.0, −13.7]	−14.3 [−14.8, −13.7]	−14.2 [−14.9, −13.5]	0.964
Global early-diastolic strain rate (s^−1^)	0.81 [0.74, 0.89]	0.70 [0.64, 0.75]^*^	0.62 [0.56, 0.67]^***^	0.62 [0.55, 0.68]^**^	<0.001
Global systolic strain rate (s^−1^)	−1.00 [−1.09, −0.92]	−0.98 [−1.06, −0.90]	−0.99 [−1.10, −0.89]	−0.97 [−1.06, −0.88]	0.979
**Regional end-systolic strain (%)**					
Anterior	−12.5 [−12.8, −12.1]	−13.0 [−13.4, −12.5]	−13.0 [−13.4, −12.5]	−13.2 [−13.9, −12.6]	0.158
Septal	−15.1 [−15.6, −14.7]	−13.7 [−14.2, −13.3]^***^	−14.1 [−14.7, −13.5]^*^	−12.9 [−13.6, −12.1]^***^	<0.001
Inferior	−12.8 [−13.1, −12.5]	−13.4 [−13.8, −13.1]	−13.5 [−13.9, −13.1]	−13.0 [−13.5, −12.5]	0.058
Lateral	−16.8 [−17.2, −16.5]	−17.7 [−18.1, −17.3]^**^	−18.1 [−18.5, −17.7]^***^	−17.9 [−18.3, −17.4]^**^	<0.001
**Radial**					
Global end-systolic strain (%)	21.9 [20.7, 23.1]	19.4 [18.0, 20.8]^*^	21.6 [20.4, 22.6]	19.7 [17.7, 21.7]	0.021
Global early-diastolic strain rate (s^−1^)	−1.62 [−1.74, −1.51]	−1.42 [−1.54, −1.30]	−1.41 [−1.52, −1.30]	−1.34 [−1.48, −1.19]^*^	0.021
Global systolic strain rate (s^−1^)	1.51 [1.39, 1.63]	1.32 [1.19, 1.47]	1.52 [1.39, 1.66]	1.34 [1.20, 1.47]	0.088
**Regional end-systolic strain (%)**					
Anterior	21.3 [20.5, 22.2]	19.0 [18.1, 20.0]^**^	20.7 [19.9, 21.5]	18.4 [16.9, 19.7]^**^	<0.001
Septal	19.9 [19.2, 20.6]	19.7 [18.9, 20.4]	21.3 [20.6, 22.0]	18.9 [17.9, 19.9]	<0.001
Inferior	23.8 [22.9, 24.8]	20.4 [19.5, 21.3]^***^	24.1 [23.1, 25.1]	18.4 [16.5, 20.3]^***^	<0.001
Lateral	24.4 [23.7, 25.0]	20.9 [20.1, 21.7]^***^	23.9 [23.3, 24.5]	21.2 [19.8, 22.6]^***^	<0.001

*Data is presented as mean [95% confidence interval]. P-value shows the one-way ANOVA test value between groups. post-hoc test by Bonferroni*:

* *p <0.05*;

** *p <0.01*;

*** *p <0.001 vs. controls*.

**Figure 4:**
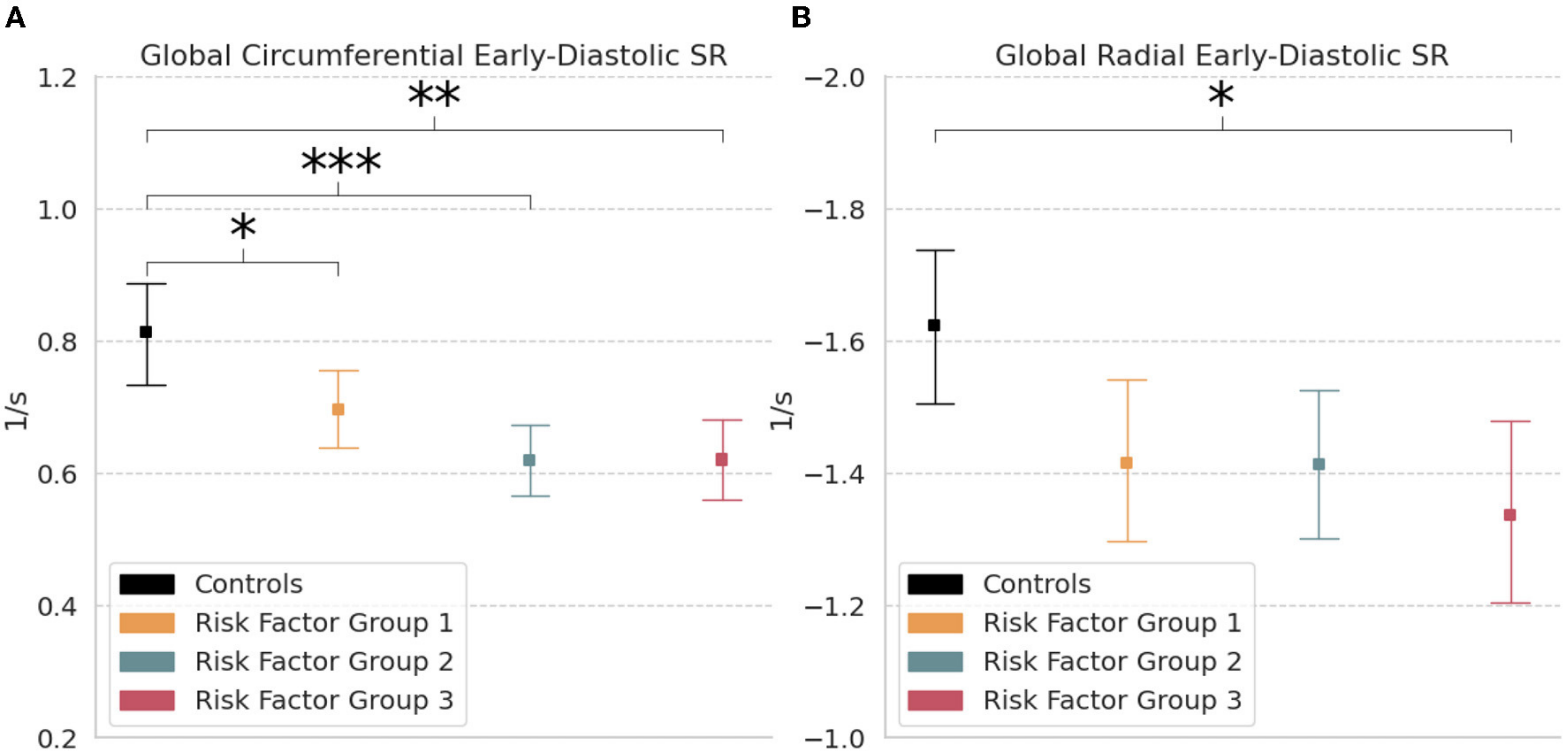
Global strain rate. Measures of **(A)** Circumferential and **(B)** Radial early-diastolic strain rate (SR) in controls and risk factor groups. Strain results are visualized as mean with 95% confidence interval. *post-hoc* test by Bonferroni: *p < 0.05; **p < 0.01; ***p < 0.001.

Global circumferential early-diastolic SR was significantly reduced in RFG1 (0.70 s^−1^ [0.64, 0.75]; *p* < 0.05), RFG2 (0.62 s^−1^ [0.56, 0.67]; *p* < 0.001), and RFG3 (0.62 s^−1^ [0.55, 0.68]; *p* < 0.01) relative to controls (0.81 s^−1^ [0.74, 0.89]) (Table 2; Figure 4A). Radial early-diastolic SR was also significantly reduced in RFG3 (−1.34 s^−1^ [−1.48, −1.19]; *p* < 0.05) compared to controls (−1.62 s^−1^ [−1.74, −1.51]) (Figure 4B). There were no significant differences between controls and RFGs in global systolic SR.

Body surface area was negatively associated with circumferential and radial end-systolic strain (*p* < 0.001; *p* < 0.001), early-diastolic SR (*p* < 0.001; *p* < 0.01), and systolic SR (*p* < 0.001; *p* < 0.01). Mean arterial pressure was negatively associated with early-diastolic circumferential SR (*p* < 0.01), while HbA1c was not significantly associated with strain measures (Table 3).

**Table 3:** Independent correlates of global strain measures.

	**End-systolic strain**		**Early-diastolic strain rate**		**Systolic strain rate**	
	**β**	***P*-value**	**β**	***P*-value**	**β**	***P*-value**
**Circumferential**						
Body surface area	−0.691	<0.001	−0.067	<0.001	−0.090	<0.001
Mean arterial pressure	−0.106	0.471	−0.046	0.007	0.020	0.383
Hemoglobin A1c	0.168	0.261	0.002	0.901	0.024	0.303
**Radial**						
Body surface area	−1.404	<0.001	−0.088	0.009	−0.112	0.003
Mean arterial pressure	0.407	0.264	−0.023	0.481	0.046	0.208
Hemoglobin A1c	−0.016	0.967	−0.013	0.691	−0.018	0.629

### Regional Strain

**Figure 5:**
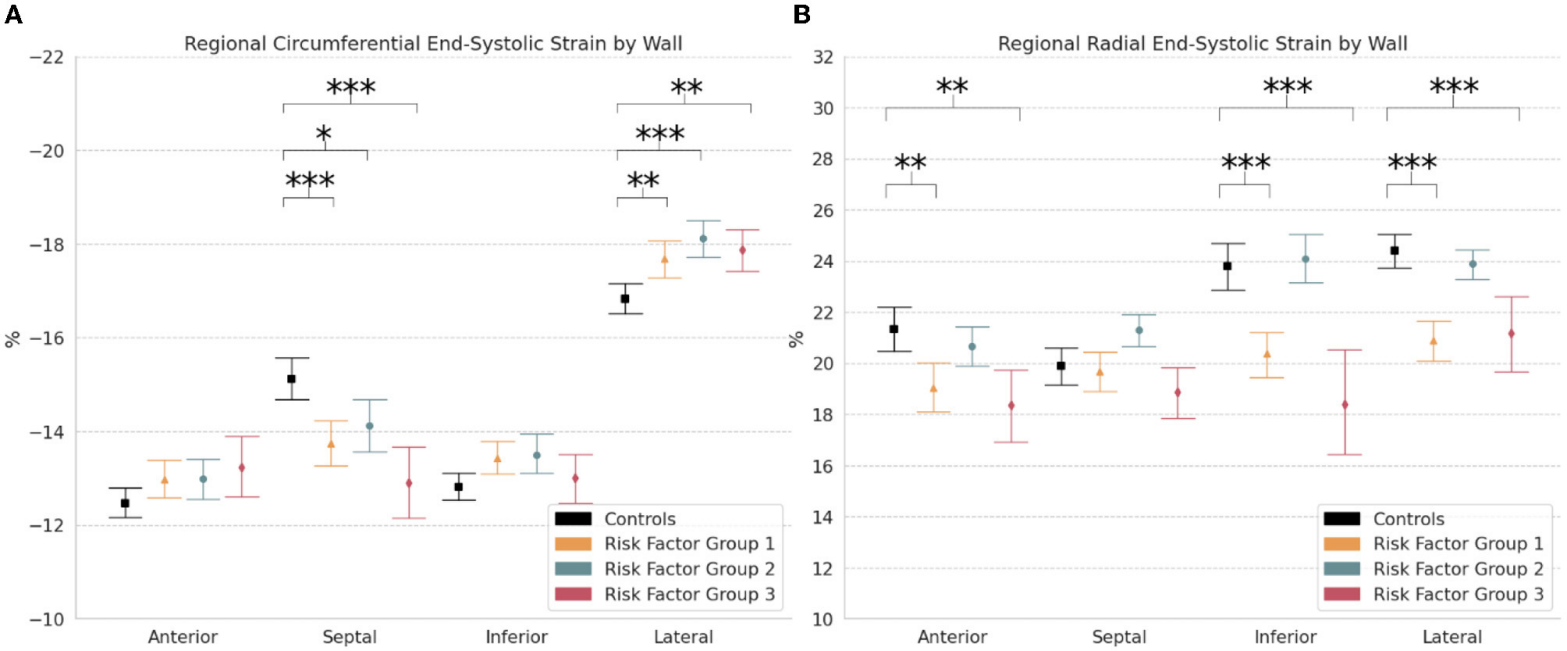
Regional strain. Measures of **(A)** Circumferential and **(B)** Radial end-systolic strain by wall (i.e., anterior, septal, inferior, and lateral) in controls and risk factor groups. Strain results are visualized as mean with 95% confidence interval. *post-hoc* test by Bonferroni: *p < 0.05; **p < 0.01; ***p < 0.001.

Regional comparisons of end-systolic strain by wall showed significantly lower circumferential strain in the septal wall of RFG1 (−13.7% [−14.2, −13.3]; *p* < 0.001), RFG2 (−14.1% [−14.7, −13.5]; *p* < 0.05), and RFG3 (−12.9% [−13.6, −12.1]; *p* < 0.001) compared to controls (−15.1% [−15.6, −14.7]) (Table 2; Figure 5A). Notably, circumferential strain was significantly increased in the lateral wall of RFG1 (−17.7% [−18.1, −17.3]; *p* < 0.01), RFG2 (−18.1% [−18.5, −17.7]; *p* < 0.001), and RFG3 (−17.9% [−18.3, −17.4]; *p* < 0.01) compared to controls (−16.8% [−17.2, −16.5]). Radial strain was decreased in the anterior (*p* < 0.01), inferior (*p* < 0.001), and lateral (*p* < 0.001) walls of RFG1 and RFG3 compared to controls (Figure 5B).

## Discussion

DeepStrain analysis of standard short-axis cine-MRI images identified evidence of ALVDD and ALVSD in asymptomatic young subjects with at least one cardiovascular risk factor including overweight, hypertension, and T2DM, but without presence or history of cardiovascular disease. Despite comparable LV mass, volumes, and ejection fraction to controls, all RFGs showed impairment of LV circumferential early-diastolic SR as well as regional circumferential end-systolic strain. On multivariate linear regression analysis, body surface area correlated negatively with all strain measures, and mean arterial pressure was a negative correlate of circumferential early-diastolic SR. Our results are consistent with similar studies in older adults with similar risk factors. However, most studies have predominantly focused on one or two risk factors groups, which makes comparisons across the three groups difficult due to differences in software and operator. Our study is the first to report reference strain values across control, overweight, hypertension, and T2DM populations using a single user-independent, fully-automatic strain analysis software.

### Asymptomatic Diastolic Dysfunction

The link from obesity, hypertension, and diabetes to diastolic dysfunction across multiple age groups has been well established in previous studies (4). For instance, a study in asymptomatic older adults recently reported a progressive decline in circumferential early-diastolic SR from controls to newly diagnosed T2DM to longstanding T2DM, and reduced radial early-diastolic SR in longstanding T2DM. This suggests asymptomatic diastolic dysfunction may occur early in the pathogenesis of T2DM, and accumulate gradually over time (13). However, data regarding diastolic dysfunction in younger (i.e., mean age <45 years old) asymptomatic populations is limited. Echocardiography studies have reported reduced circumferential early-diastolic SR in obesity (8), and similar reductions have been documented in obese subjects using tagging-MRI (24). In this cine-MRI study, we showed that a significant reduction already exists in overweight subjects (i.e., RFG1), even when a lower BMI threshold of 25 kg/m^2^ was used. Further, we found that both circumferential and radial early-diastolic SR were inversely associated with body surface area, which is an important finding because body surface area is a prognostic indicator of adverse outcomes in heart failure. In hypertensive subjects without diabetes, reduction in circumferential early-diastolic SR values proportional to standard echocardiography grading of diastolic function has also been reported (25). Similarly, our study showed these values were reduced in RFG2 relative to controls, and were inversely associated with mean arterial pressure. Taking both overweight and hypertension into consideration, these results are consistent with the observed benefits of weight reduction, namely reduced mean arterial pressure and improved diastolic parameters. In addition, since both obesity and hypertension are strong risk factors for T2DM, it is not surprising that circumferential and radial early-diastolic SR were significantly reduced in RFG3 compared to controls, which is consistent with previous studies in T2DM (8, 24). These results are evidence of ALVDD in young adults with overweight, hypertension, and T2DM, characterized by a progressive deterioration in early-diastolic SR from controls to RFG1 to RFG2 to RFG3. Thus, DeepStrain could become a useful tool in the evaluation of suspected diastolic dysfunction.

### Asymptomatic Systolic Dysfunction

Multiple studies in older adults have reported reduced systolic strain with preserved ejection fraction in obesity, hypertension, and T2DM (10, 14, 15, 24, 26). For example, cardiac MRI studies measuring global circumferential end-systolic strain have reported a clear reduction in obesity (14), and progressive decline from controls to hypertension to hypertension with T2DM (15). Evidence of reduced systolic strain in younger asymptomatic populations is less clear. Studies in obese subjects have shown no significant differences in measures of global circumferential end-systolic strain relative to lean controls (8, 24, 27). Likewise, we found global circumferential end-systolic strain was preserved in overweight subjects relative to lean controls, but was negatively associated with increased body surface area. Our study showed radial and circumferential global strain values were preserved in RFG2 subjects, but were associated with increased mean arterial pressure. Conflicting results have been reported in T2DM. Haley et al. showed global circumferential end-systolic strain values were reduced in T2DM subjects relative to non-diabetic lean controls (8), whereas a more recent but smaller study by Khan et al. reported no significant differences in values between controls and T2DM subjects (24). We also found comparable values between controls and RFG3 subjects, and no association between strain and HbA1c levels, the latter consistent with the finding that HbA1c is not always associated with LV dysfunction. These discrepancies may be caused by differences between echocardiography, tagging- and cine-MRI imaging modalities, which could be exacerbated when differences in strain are less pronounced. With some exceptions where the mean age was 43 years old (26), studies in younger populations have not reported radial strain, and to the best of our knowledge none of them has presented regional evaluations.

The typical progression from stage A heart failure (i.e., inclusion into a RFG) to symptomatic stage C heart failure begins with ALVDD, followed by ALVSD, overt systolic dysfunction, and finally by symptomatic heart failure (7, 9). Although the presence of ALVDD in all RFGs was clearly indicated by a reduction in global early-diastolic SR, the use of global systolic strain measures to establish the presence or absence of ALVSD was less conclusive in both our study and previous studies. Indeed, our finding that global circumferential strain is preserved may be explained by the small sample sizes and inability to achieve statistical significance in *post-hoc* analyses, but could also imply that global measures are less sensitive to subtle systolic dysfunction than regional measures, as previously shown for mild hypertension (28). In fact, accurate evaluation of regional strain is challenging but potentially very important as it could provide additional insights regarding early disease processes. For instance, asymmetric septal hypertrophy due to increased afterload has been considered a consequence of increased wall stress on the septum compared to the free lateral wall. This asymmetric response to stress results in reduced strain values in the septal wall and increased values in the lateral as reported in human and animal studies, which could be explained by a larger compensatory preload recruitment in the free wall compared to the septum (28, 29). Similar asymmetric strain alterations have been reported in patients with non-ischemic cardiomyopathy, and in these patients partial left ventriculectomy has been shown to normalize wall stress and improve septal strain (30). Further, septal-only fibrosis in non-ischemic cardiomyopathy has been documented to result in worse global circumferential strain relative to patients with free-wall only fibrosis, highlighting the need to identify both location and extent of deformation abnormalities (31). In line with these studies, we found significant regional alterations in circumferential end-systolic strain in asymptomatic young adults with overweight, hypertension, and T2DM, characterized by reduced septal strain and increased lateral strain. Thus, our regional analysis with DeepStrain provides evidence of the existence of heterogeneous strain alterations early in the disease process, although further studies are needed to assess regional strain alterations in non-ischemic patient populations.

### DeepStrain-Based Analysis

To our best knowledge, the current study is the first to apply an end-to-end deep learning-based workflow for myocardial strain analysis detection of ALVDD and ALVSD in risk populations, an approach that offers several advantages over traditional techniques. Firstly, there are no explicit assumptions about myocardial tissue properties imbedded into the model (e.g., incompressible myocardium), which is common in conventional methods (32), while this might not always accurately reflect the underlying biomechanical motion (33). Secondly, analysis per subject was done two orders of magnitude faster than conventional methods (32). Thirdly, the open-source nature of the software enables the scientific community to easily apply our approach to other populations. Lastly, the purchase of commercially available software licenses whose accuracy depends on the segmentation procedure and vendor is not always an option for research groups (16), whereas open-source software provides key opportunities for cost reduction, clinical engagement in software development, and accelerated innovation.

The results presented in this study were based on cine-MRI images acquired using a 3.0 T MRI scanner with a custom 60-element coil. However, DeepStrain could potentially capture similar abnormalities using other clinical scanners. For instance, DeepStrain was trained using images acquired using both 1.5 T and 3.0 T MRI scanners (23), therefore our method could be applied to data acquired with both magnetic field strengths. Further, we previously demonstrated that our method can be applied to images collected with a different vendor using tagging-MRI as reference (32). In addition, we have reported high intra-scanner repeatability in strain measures in data acquired with a 6-channel body surface coil (18), therefore a custom 60-element coil might not be necessary to capture subclinical abnormalities.

The DeepStrain model used in this study was based on five populations covering a wide spectrum of cardiac anatomy and function, which reduces the bias toward a single heart condition. Specifically, the training cohort included groups with dilated and hypertrophic cardiomyopathy, myocardial infarction, and abnormal right ventricle. Obesity and obesity-related comorbidities often result in structural abnormalities such as LV hypertrophy, potentially leading to obesity cardiomyopathy, hypertensive heart disease or diabetic cardiomyopathy. Thus, the inclusion of the cardiomyopathy cohorts during training was particularly important. Nevertheless, in this study, the RFGs showed normal anatomy compared to the control group based on measures of volume and mass. Therefore, further validation in patients with more advanced cardiac diseases is necessary and might require additional training. In addition, validation in larger cohorts and comparisons to commercial software are necessary to further demonstrate the clinical utility of DeepStrain.

### Study Limitations

DeepStrain currently does not support long-axis images, therefore longitudinal strain was not reported. From a technical perspective, a joint model that uses both short and long axis images would be needed to enforce consistency in the evaluated deformation. For instance, such model could be based on recently published cine-MRI data with both long and short axis views (34). The small participant numbers in the T2DM subgroup may hinder the reliability of the measured strain, especially for regional strain analysis. Since this was a cross-sectional study, we could not monitor the progression of subclinical dysfunction or link these findings to clinical outcomes. Further, two of the groups included in the study cohort had a mixture of risk factors, therefore the current study cannot separate the independent impact of each of the three risk factors.

## Conclusions

In young adults with overweight, hypertension, and T2DM risk factors, early-diastolic SR suggested ALVDD, and regional alterations in end-systolic strain indicated ALVSD despite preserved ejection fraction. These results demonstrate that the application of DeepStrain in routine cardiac MRI studies would fully-automatically offer useful information about the underlying biomechanical motion using standard short-axis cine-MRI data.

## Data Availability Statement

The raw data supporting the conclusions of this article will be made available by the authors, without undue reservation.

## Ethics Statement

The studies involving human participants were reviewed and approved by Medical Ethical Committee of the University Medical Center Groningen (No. 2016/476). The participants provided their written informed consent to participate in this study.

## Author Contributions

MM contributed with implementation and analysis of strain methodology. GS, MB, and NP contributed with data acquisition. MM and GS analysed and interpreted that data and drafted the manuscript. All other authors revised the drafted manuscript, contributed critical intellectual content, and approved the final version of the manuscript.

## Funding

This work was supported by the Dutch Heart Association (2016T042) and in part by the U.S. National Cancer Institute under Grant 1R01CA218187-01A1.

## Conflict of Interest

The authors declare that the research was conducted in the absence of any commercial or financial relationships that could be construed as a potential conflict of interest.

## Publisher's Note

All claims expressed in this article are solely those of the authors and do not necessarily represent those of their affiliated organizations, or those of the publisher, the editors and the reviewers. Any product that may be evaluated in this article, or claim that may be made by its manufacturer, is not guaranteed or endorsed by the publisher.

## Abbreviations

ALVDD: asymptomatic left ventricular diastolic dysfunction
ALVSD: asymptomatic left ventricular systolic dysfunction
ANOVA: analysis of variance
BMI: body mass index
MRI: magnetic resonance imaging
HbA1c: hemoglobin A1c
LV: left ventricular
RFG: risk factor group
SR: strain rate
T2DM: type 2 diabetes mellitus.

## References

[1] Afshin A Sur PJ Fay KA Cornaby L Ferrara G Salama JS. Health effects of dietary risks in 195 countries, 1990–2017: a systematic analysis for the Global Burden of Disease Study 2017. Lancet. (2019) 393:1958–72. 10.1016/S0140-6736(19)30041-8 30954305 PMC6899507

[2] Guthold R Stevens GA Riley LM Bull FC. Worldwide trends in insufficient physical activity from 2001 to 2016: a pooled analysis of 358 population-based surveys with 1·9 million participants. Lancet Glob Heal. (2018) 6:e1077–86. 10.1016/S2214-109X(18)30357-7 30193830

[3] Petrie JR Guzik TJ Touyz RM. Diabetes, hypertension, and cardiovascular disease: clinical insights and vascular mechanisms. Can J Cardiol. (2018) 34:575–84. 10.1016/j.cjca.2017.12.005 29459239 PMC5953551

[4] Mazloomzadeh S Zarandi FK Shoghli A Dinmohammadi H. Metabolic syndrome, its components and mortality: a population-based study. Med J Islam Repub Iran. (2019) 33:11. 10.47176/mjiri.33.11 31086790 PMC6504944

[5] von Jeinsen B Vasan RS McManus DD Mitchell GF Cheng S Xanthakis V. Joint influences of obesity, diabetes, and hypertension on indices of ventricular remodeling: Findings from the community-based Framingham Heart Study. PLoS One. (2020) 15:e0243199. 10.1371/journal.pone.0243199 33301464 PMC7728232

[6] Christiansen MN Køber L Weeke P Vasan RS Jeppesen JL Smith JG. Age-specific trends in incidence, mortality, and comorbidities of heart failure in Denmark, 1995 to 2012. Circulation. (2017) 135:1214–23. 10.1161/CIRCULATIONAHA.116.025941 28174193

[7] Kosmala W Marwick TH. Asymptomatic left ventricular diastolic dysfunction: predicting progression to symptomatic heart failure. JACC Cardiovasc Imaging. (2020) 13:215–27. 10.1016/j.jcmg.2018.10.039 31005530

[8] Haley JE Zhiqian G Philip KR Nicolas ML Thomas KR Lawrence DM. Reduction in myocardial strain is evident in adolescents and young adults with obesity and type 2 diabetes. Pediatr Diabet. (2020) 21:243–50. 10.1111/pedi.12961 31825129

[9] Sara JD Toya T Taher R Lerman A Gersh B Anavekar NS. asymptomatic left ventricle systolic dysfunction. Eur Cardiol Rev. (2020) 15:e13. 10.15420/ecr.2019.14 32373186 PMC7199190

[10] Ng ACT Bertini M Ewe SH van der Velde ET Leung DY Delgado V. Defining subclinical myocardial dysfunction and implications for patients with diabetes mellitus and preserved ejection fraction. Am J Cardiol. (2019) 124:892–898. 10.1016/j.amjcard.2019.06.011 31375242

[11] Uppot RN. Technical challenges of imaging & image-guided interventions in obese patients. Br J Radiol. (2018) 91:20170931. 10.1259/bjr.20170931 29869898 PMC6223172

[12] Shah RV Abbasi SA Kwong RY. Role of cardiac MRI in diabetes. Curr Cardiol Rep. (2014) 16:449. 10.1007/s11886-013-0449-0 24430012 PMC3965673

[13] Liu X Yang ZG Gao Y Xie LJ Jiang L Hu BY. Left ventricular subclinical myocardial dysfunction in uncomplicated type 2 diabetes mellitus is associated with impaired myocardial perfusion: a contrast-enhanced cardiovascular magnetic resonance study. Cardiovasc Diabetol. (2018) 17:139. 10.1186/s12933-018-0782-0 30373588 PMC6206833

[14] Homsi R Yuecel S Schlesinger-Irsch U Meier-Schroers M Kuetting D Luetkens J. Epicardial fat, left ventricular strain, and T1-relaxation times in obese individuals with a normal ejection fraction. Acta radiol. (2019) 60:1251–7. 10.1177/0284185119826549 30727747

[15] Kropidlowski C Meier-Schroers M Kuetting D Sprinkart A Schild H Thomas D. CMR based measurement of aortic stiffness, epicardial fat, left ventricular myocardial strain and fibrosis in hypertensive patients. IJC Hear Vasc. (2020) 27:100477. 10.1016/j.ijcha.2020.100477 32099896 PMC7026624

[16] Lim C Blaszczyk E Riazy L Wiesemann S Schüler J von Knobelsdorff-Brenkenhoff F. Quantification of myocardial strain assessed by cardiovascular magnetic resonance feature tracking in healthy subjects—influence of segmentation and analysis software. Eur Radiol. (2021) 31:3962–72. 10.1007/s00330-020-07539-5 33277669 PMC8128822

[17] Morales MA Izquierdo-Garcia D Aganj I Kalpathy-Cramer J Rosen BR Catana C. Implementation and validation of a three-dimensional cardiac motion estimation network. Radiol Artif Intell. (2019) 1:e180080. 10.1148/ryai.2019180080 32076659 PMC6677286

[18] Morales MA van den Boomen M Nguyen C Kalpathy-Cramer J Rosen BR Stultz CM. DeepStrain: a deep learning workflow for the automated characterization of cardiac mechanics. Front Cardiovasc Med. (2021) 8:730316. 10.3389/fcvm.2021.730316 34540923 PMC8446607

[19] Wong CM Hawkins NM Jhund PS Macdonald MR Solomon SD Granger CB. Clinical characteristics and outcomes of young and very young adults with heart failure: the CHARM programme (candesartan in heart failure assessment of reduction in mortality and morbidity). J Am Coll Cardiol. (2013) 62:1845–54. 10.1016/j.jacc.2013.05.072 23850914

[20] Lloyd-Jones DM Larson MG Leip EP Beiser A D'Agostino RB Kannel WB. Lifetime risk for developing congestive heart failure: the framingham heart study. Circulation. (2002) 106:3068–72. 10.1161/01.CIR.0000039105.49749.6F 12473553

[21] Prakken NH Velthuis BK Teske AJ Mosterd A Mali WP Cramer MJ. Cardiac MRI reference values for athletes and nonathletes corrected for body surface area, training hours/week and sex. Eur J Cardiovasc Prev Rehabil. (2010) 17:198–203. 10.1097/HJR.0b013e3283347fdb 20042862

[22] Cerqueira MD Weissman NJ Dilsizian V Jacobs AK Kaul S Laskey WK. standardized myocardial segmentation and nomenclature for tomographic imaging of the heart: a statement for healthcare professionals from the cardiac imaging committee of the council on clinical cardiology of the American heart association. Circulation. (2002) 105:539–42. 10.1161/hc0402.102975 11815441

[23] Bernard O Lalande A Zotti C Cervenansky F Yang X Heng PA. Deep learning techniques for automatic mri cardiac multi-structures segmentation and diagnosis: is the problem solved? IEEE Trans Med Imaging. (2018) 37:2514–25. 10.1109/TMI.2018.2837502 29994302

[24] Khan JN Wilmot EG Leggate M Singh A Yates T Nimmo M. Subclinical diastolic dysfunction in young adults with Type 2 diabetes mellitus: a multiparametric contrast-enhanced cardiovascular magnetic resonance pilot study assessing potential mechanisms. Eur Heart J Cardiovasc Imaging. (2014) 15:1263–9. 10.1093/ehjci/jeu121 24970723

[25] Sharif H Ting S Forsythe L McGregor G Banerjee P O'Leary D. Layer-specific systolic and diastolic strain in hypertensive patients with and without mild diastolic dysfunction. Echo Res Pract. (2018) 5:41–9. 10.1530/ERP-17-0072 29432196 PMC5827572

[26] Liu H Wang J Pan Y Ge Y Guo Z Zhao S. Early and quantitative assessment of myocardial deformation in essential hypertension patients by using cardiovascular magnetic resonance feature tracking. Sci Rep. (2020) 10:3582. 10.1038/s41598-020-60537-x 32107428 PMC7046638

[27] Xu E Kachenoura N Della Valle V Dubern B Karsenty A Tounian P. Multichamber dysfunction in children and adolescents with severe obesity: a cardiac magnetic resonance imaging myocardial strain study. J Magn Reson Imaging. (2021) 54:1393–1403. 10.1002/jmri.27796 34155711

[28] Baltabaeva A Marciniak M Bijnens B Moggridge J He FJ Antonios TF. Regional left ventricular deformation and geometry analysis provides insights in myocardial remodelling in mild to moderate hypertension. Eur J Echocardiogr. (2008) 9:501–8. 10.1016/j.euje.2007.08.004 17905662

[29] Murai D Yamada S Hayashi T Okada K Nishino H Nakabachi M. Relationships of left ventricular strain and strain rate to wall stress and their afterload dependency. Heart Vessels. (2017) 32:574–83. 10.1007/s00380-016-0900-4 27734145

[30] Young AA Dokos S Powell KA Sturm B McCulloch AD Starling RC. Regional heterogeneity of function in nonischemic dilated cardiomyopathy. Cardiovasc Res. (2001) 49:308–18. 10.1016/S0008-6363(00)00248-0 11164841

[31] Csecs I Pashakhanloo F Paskavitz A Jang J Al-Otaibi T Neisius U. Association between left ventricular mechanical deformation and myocardial fibrosis in nonischemic cardiomyopathy. J Am Heart Assoc. (2020) 9:e016797. 10.1161/JAHA.120.016797 33006296 PMC7792406

[32] Tobon-Gomez C De Craene M McLeod K Tautz L Shi W Hennemuth A. Benchmarking framework for myocardial tracking and deformation algorithms: an open access database. Med Image Anal. (2013) 17:632–648. 10.1016/j.media.2013.03.008 23708255

[33] Kumar V Ryu AJ Manduca A Rao C Gibbons RJ Gersh BJ. Cardiac MRI demonstrates compressibility in healthy myocardium but not in myocardium with reduced ejection fraction. Int J Cardiol. (2021) 322:278–83. 10.1016/j.ijcard.2020.08.087 32871188

[34] Campello VM Gkontra P Izquierdo C Martin-Isla C Sojoudi A Full PM. Multi-Centre, multi-vendor and multi-disease cardiac segmentation: the m&ms challenge. IEEE Trans Med Imaging. (2021) 40:3543–54. 10.1109/TMI.2021.3090082 34138702

