# Supplementary material for: DeepStrain Evidence of Asymptomatic Left Ventricular Diastolic and Systolic Dysfunction in Young Adults With Cardiac Risk Factors
Source: Front Cardiovasc Med. 2022 Apr 11;9:831080. doi: 10.3389/fcvm.2022.831080 (PMC9035693; doi:10.3389/fcvm.2022.831080)
Supplement: Supplementary file 1 [file Table_1.DOCX]

Supplementary Material

# Supplementary Table

**Supplementary Table 1** Left ventricular parameters of controls and risk factor groups (RFG).

|  | **Control** | **RFG 1** | **RFG 2** | **RFG 3** | ***P*-value** |
| --- | --- | --- | --- | --- | --- |
| **End-diastolic mass (g)** | 111 ± 24 | 119 ± 24 | 128 ± 28 | 123 ± 29 | 0.098 |
| **End-diastolic volume (ml)** | 176 ± 30 | 166 ± 31 | 166 ± 39 | 151 ± 34 | 0.091 |
| **End-systolic volume (ml)** | 68 ± 15 | 62 ± 19 | 63 ± 19 | 56 ± 15 | 0.167 |
| **Ejection fraction (%)** | 61 ± 5 | 62 ± 5 | 61 ± 5 | 62 ± 5 | 0.641 |

Data reported as mean ± standard deviation.

*P*-value shows the one-way ANOVA test value between groups.
